# Mechanistic insights into the dual role of CCAR2/DBC1 in cancer

**DOI:** 10.1038/s12276-023-01058-1

**Published:** 2023-08-01

**Authors:** Hwa Jin Kim, Sue Jin Moon, Jeong Hoon Kim

**Affiliations:** 1 Department of Health Sciences and Technology, Samsung Advanced Institute for Health Sciences and Technology, Sungkyunkwan University, Seoul, 06351 South Korea; 2 Research Institute for Future Medicine, Samsung Medical Center, Seoul, 06351 South Korea

**Keywords:** Transcription, Oncogenes, Epigenetics

## Abstract

Cell cycle and apoptosis regulator 2 (CCAR2), also known as deleted in breast cancer 1 (DBC1), has been recently identified as a master regulator of transcriptional processes and plays diverse roles in physiology and pathophysiology, including as a regulator of apoptosis, DNA repair, metabolism, and tumorigenesis. CCAR2 functions as a coregulator of various transcription factors and a critical regulator of numerous epigenetic modifiers. Based on its ability to stimulate apoptosis by activating and stabilizing p53, CCAR2 was initially considered to be a tumor suppressor. However, an increasing number of studies have shown that CCAR2 also functions as a tumor-promoting coregulator by activating oncogenic transcription factors and regulating the enzymatic activity of epigenetic modifiers, indicating that CCAR2 may play a dual role in cancer progression by acting as a tumor suppressor and tumor promoter. Here, we review recent progress in understanding the dual tumor-suppressing and oncogenic roles of CCAR2 in cancer. We discuss CCAR2 domain structures, its interaction partners, and the molecular mechanisms by which it regulates the activities of transcription factors and epigenetic modifiers.

## Cancer: nuclear protein both an off- and on-switch for tumors

A nuclear protein, originally identified by its tumor-suppressing activity, has been shown to also be a tumor promoter. CCAR2 promotes programmed cell death, suppressing tumors by triggering the removal of damaged cells. However, recent research, reviewed by Jeong Hoon Kim at Sungkyunkwan University, Seoul, South Korea, and co-workers, shows that CCAR2’s role in cancer is more complex than first thought. CCAR2 has a complicated shape that allows it to perform many functions, including regulating proteins that reorganize DNA architecture to expose or hide genes, activating or deactivating them, and thereby suppressing or promoting cancer depending on the genes involved. Increased levels of CCAR2 are associated with poor prognosis, indicating its potential for use as a prognostic marker. Understanding CCAR2’s dual nature may help in developing new treatments for various cancers.

## Introduction

Gene transcription is a complex process orchestrated by numerous transcription factors (TFs), coregulators, and epigenetic modifiers^1,2^. TFs control gene expression by binding to specific regulatory DNA sequences called enhancers and recruiting a series of coregulators to their target genes. Transcriptional coregulators, which include coactivators and corepressors, associate with TFs directly or indirectly as components of multiprotein complexes and transmit and integrate cell signals by functioning as scaffolds to coordinate the assembly of transcription complexes and/or by tethering various posttranslational modifying enzymes to specific transcription sites^1,2^. Epigenetic modifiers function as components of coregulator complexes and are enzymes that alter chromatin accessibility to regulate gene expression by posttranslationally modifying histones and nonhistone proteins such as TFs and coregulators.

Cell cycle and apoptosis regulator 2 (CCAR2), also known as deleted in breast cancer 1 (DBC1) or KIAA1967, was originally discovered more than two decades ago and cloned from a human chromosome 8p21 region, which had been homozygously deleted in a subset of breast cancers^3^. This genomic region contains four identified genes and two previously uncharacterized open reading frames, and these genes with unknown functions were named DBC1 and DBC2. However, a study showed that, in contrast to DBC2, DBC1 was expressed in most breast tumors^3^, and subsequent studies showed that DBC1 was frequently overexpressed in breast cancer and other malignancies^4–6^. Therefore, considering its function and homology to its paralog CCAR1, DBC1 has been officially renamed CCAR2. CCAR2 has been recently identified as a critical regulator of transcriptional processes mediated through its regulatory function in conjunctions with TFs, such as p53^7,8^, estrogen receptor α (ERα)^9–11^, and androgen receptor (AR)^12,13^, and with epigenetic modifiers, including SIRT1^5,7–9,14^, histone deacetylase 3 (HDAC3)^15,16^, SUV39H1^17,18^, lysine methyltransferase 2D (KMT2D)^19^, and p300/CREB-binding protein (CBP)^19^.

CCAR2 has been reported to play physiological and pathological roles in a variety of cellular processes, including apoptosis, DNA repair, aging, metabolism, circadian clocks, cell proliferation, and tumorigenesis, and has been identified as a potential therapeutic target in multiple types of cancer^6,20–24^. In this review, we discuss recent advances in our understanding of the role and mechanism of CCAR2 as a key coregulator of TFs and epigenetic modifiers, paying special attention given to the dual role of CCAR2 as a tumor suppressor and tumor promoter during tumorigenesis.

## Domain structures and interaction partners of CCAR2

**Fig. 1:**
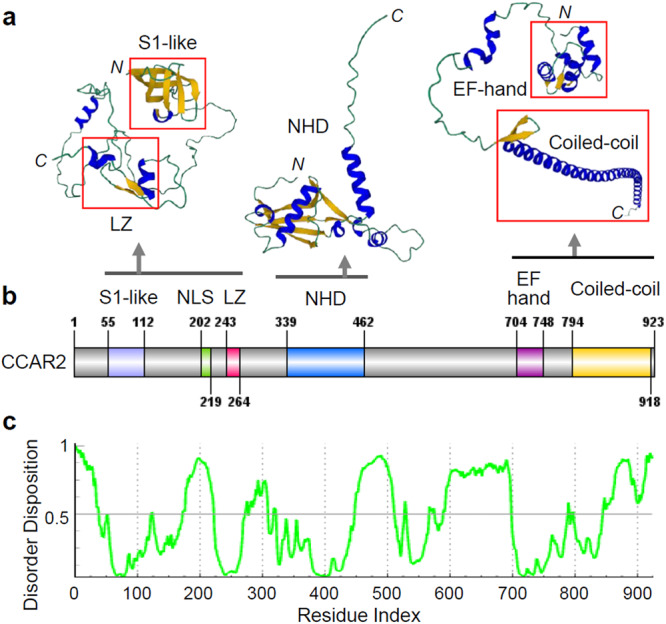
Domain structure of the CCAR2 protein. **a** AlphaFold2 prediction of the functional domains of CCAR2 using ColabFold software (https://colabfold.mmseqs.com). Ribbon representation of the predicted S1-like RNA-binding domain (S1-like), leucine zipper motif (LZ), nudix homology domain (NHD), EF-hand, and a coiled-coil domain. α-Helix, blue; β-sheet, yellow; intrinsically disordered region, green. **b** Schematic representation of the domain organization of CCAR2. NLS, nuclear localization signal. **c** Intrinsically disordered regions of CCAR2. The prediction was performed using PONDR-FIT (http://original.disprot.org/pondr-fit.php). PONDR-FIT score (y-axis) and amino acid position (x-axis) are shown. A score higher than 0.5 indicates disorder.

CCAR2 is a nuclear multidomain protein of 923 amino acids and has six major functional domains, an S1-like RNA-binding domain, a nuclear localization signal (NLS), a leucine zipper (LZ) motif, a Nudix homology domain (NHD), an EF-hand domain, and a coiled-coil domain (Fig. 1a, b). This complex domain architecture suggests that CCAR2 plays a critical role in various cellular processes, including transcriptional regulation, posttranslational modifications (PTMs), calcium signaling, and RNA recognition and processing. The AlphaFold2-predicted 3D structures of the functional domains^25,26^, including the S1-like, LZ, NHD, EF-hand, and coiled-coil domains, indicated that they are highly structured and thus may exhibit limited flexibility (Fig. 1a), and other regions, constituting more than 40% of the CCAR2 residues, are highly intrinsically disordered (Fig. 1c), suggesting a high degree of conformational flexibility and possible involvement in the formation of phase-separated bodies such as nuclear speckles^27,28^. Because intrinsically disordered regions in proteins are considered to be essential for the repertoire of protein functions, including protein‒protein interactions and liquid‒liquid phase separation^29–31^, these regions need to be further investigated because it is important to understand the dynamics of CCAR2 and its biological functions.

CCAR2 functions as a transcriptional coregulator through its interaction with various TFs, epigenetic modifiers, and other cellular proteins, including other coregulators (Table 1). The N-terminal domain (NTD) of CCAR2, which includes the S1-like domain, NLS, and LZ motif, is required for its binding to TFs, coregulators, and epigenetic modifiers, such as ERα^9,10^, ERβ^32^, AR^12,13^, COUP-TF1^33^, liver X receptor α (LXRα)^34^, nuclear factor κB (NF-κB)^35^, CCAR1^9^, β-catenin^14^, BRCA1^36^, SIRT1^7–9^, HDAC3^15^, SUV39H1^17^, KMT2D^19^, p300/CBP^19^, hMOF^37^, and checkpoint kinase 2 (CHK2)^38^. Therefore, the NTD of CCAR2 functions as a platform through which the protein binds with most, if not all, of the binding partners identified so far.

**Table 1:** CCAR2-interacting proteins and the effect of CCAR2 on their activity.

	Type	Enzyme	Effect	Ref
**Epigenetic modifiers (Coregulators)**	**HDAC**	**SIRT1**	Inhibition	^5,7–9,14,48^
		**HDAC3**	Inhibition	^15,16^
	**HMT**	**SUV39H1**	Inhibition	^17,18^
		**KMT2D**	Activation	^19^
	**E3/E4 ligase**	**MDM2**	Inhibition	^67^
		**CHIP**	Inhibition	^13^
		**CBP**	Inhibition	^71^
	**HAT**	**p300/CBP**	Activation	^19^
		**MOF**	Not determined	^37^
	**PARP**	**PARP1**	Inhibition	^45^
	**Kinase**	**CHK2**	Activation/Inhibition	^38,63^
		**IKKβ**	Activation	^61^
		**IKKα**	Not determined	^35^
		**Aurora B**	Activation	^64^
	**Type**	**Coregulator**	**Effect**	**Ref**
**Coregulators**	**Coactivator**	**β-catenin**	Activation	^14^
		**NIF1**	Activation	^88^
		**CCAR1**	Activation	^9^
		**Ajuba**	Activation	^11^
		**BRCA1**	Repression	^36^
	**Corepressor**	**BRMS1**	Repression	^54^
		**NCOR1**	Repression	^33^
	**Type**	**TF**	**Effect**	**Ref**
**Transcription factors**	**NR**	**ERα**	Activation/Stabilization	^9,10^
		**AR**	Activation/Stabilization	^12,13^
		**AR-V7**	Activation/Stabilization	^13^
		**COUP-TF1**	Activation	^33^
		**Rev-Erbα**	Activation	^46^
		**RARα**	Activation	^88^
		**ERβ**	Repression	^32^
		**LXRα**	Repression	^34^
	**Other TFs**	**p53**	Activation/Stabilization	^7,8,67,71^
		**FOXO3a**	Activation	^7^
		**c-MYC**	Activation	^85^
		**MEF2D**	Activation	^15^
		**PEA3/ETV4**	Activation	^5^
		**PROX1**	Activation	^14^
		**NF-kB/RelA**	Activation	^61,89^
		**NF-kB/RelB**	Repression	^35^
	**Type**	**Protein**	**Effect**	**Ref**
**Other proteins**		**RNA Pol II**	Elongation/Splicing	^40^
		**ZNF326**	Elongation/Splicing	^40^
		**ELL**	Stabilization	^16^
		**HSP60**	Anti-apoptosis	^100,101^
		**HNRNPL**	Nuclear body formation	^27,28^
**RNAs**	**mRNA**	**mRNAs**	Elongation/Splicing	^40^
	**LncRNA**	**MALAT1**	Repression	^42^

A structure prediction study identified the S1-like RNA-binding domain in the NTD of CCAR2 and suggested that CCAR2 might interact with RNA^39^. Interestingly, a proteomic study identified DBIRD, a protein complex containing CCAR2 and ZNF326, which regulates alternative mRNA splicing at a subset of A/T-rich exon-intron junctions^40^. CCAR2 binds directly to RNA Polymerase II (Pol II) and mRNAs and helps to integrate transcription elongation with alternative mRNA splicing. Another proteomic study also revealed that CCAR2 associates with multiple components of the spliceosome machinery^41^. These results suggest the possibility that CCAR2 may play a role in the integration of transcription with alternative mRNA splicing. The S1-like RNA-binding domain of CCAR2 may play a critical role in recognizing splicing sites in pre-mRNAs, but further investigation is needed to verify whether the S1-like domain of CCAR2 is directly involved in CCAR2 binding to mRNAs and small nuclear RNAs. In addition, the NTD of CCAR2 has been reported to bind to MALAT1 (metastasis-associated lung adenocarcinoma transcript 1), a long noncoding RNA (lncRNA)^42^. Interestingly, CCAR2 is proteolytically processed in quiescent cells, producing an N-terminally truncated CCAR2 (DN-DBC1) lacking the S1-like domain^43^. DN-DBC1 cannot bind SIRT1 and is rapidly cleared and replaced by CCAR2 when quiescent cells re-enter the cell cycle, suggesting that CCAR2 loses its inhibitory function on SIRT1 in cells in a quiescent state. Further investigation is needed to determine whether the loss of the S1-like domain in CCAR2 has important implications for its RNA-binding activity and function.

The Nudix (nucleoside diphosphate linked to some other moiety X) hydrolase family is a group of enzymes that bind and hydrolyze nucleoside diphosphate derivatives such as nicotinamide adenine dinucleotide (phosphate) (NAD(P)) and ADP-ribose^44^. They harbor a catalytic site characterized by a conserved cassette comprising 23 amino acids (Nudix box) GX_5_EX_5_AXRX_4_EXGU (U, bulky hydrophobic; X, any amino acid)^44^. The NHD of CCAR2 is likely to be catalytically inactive because the key acidic residues are not conserved in the active site motif. However, the conservation of an arginine residue in the Nudix box suggests that this residue might stabilize the substrate-binding pocket, and CCAR2-NHD has been postulated to bind nucleoside diphosphate derivatives^39^. Indeed, the NHD of CCAR2 binds to NAD+, and its binding to NAD+ leads to the negative regulation of the CCAR2 interaction with poly(ADP-ribose) polymerase 1 (PARP1)^45^. Atomic-resolution homology modeling based on five known crystal structures of Nudix domain proteins showed that NAD+ can bind CCAR2-NHD, and this result was confirmed via biochemical binding assays, suggesting that CCAR2-NHD functions as a NAD+-binding domain^45^. The CTD of CCAR2, including the EF-hand and coiled-coil domains, interacts with Rev-erbα, a nuclear receptor that is critical for integrating circadian rhythms with metabolism and differentiation^46^. Through these multiple functional domains, CCAR2 can interact with and regulate the function of a variety of proteins involved in cellular processes such as transcription, epigenetic regulation, apoptosis, DNA repair, metabolism, and circadian rhythms^6,20–24^.

## CCAR2 as a key regulator of epigenetic modifiers

### Negative regulation of SIRT1, HDAC3, and SUV39H1 by CCAR2

**Fig. 2:**
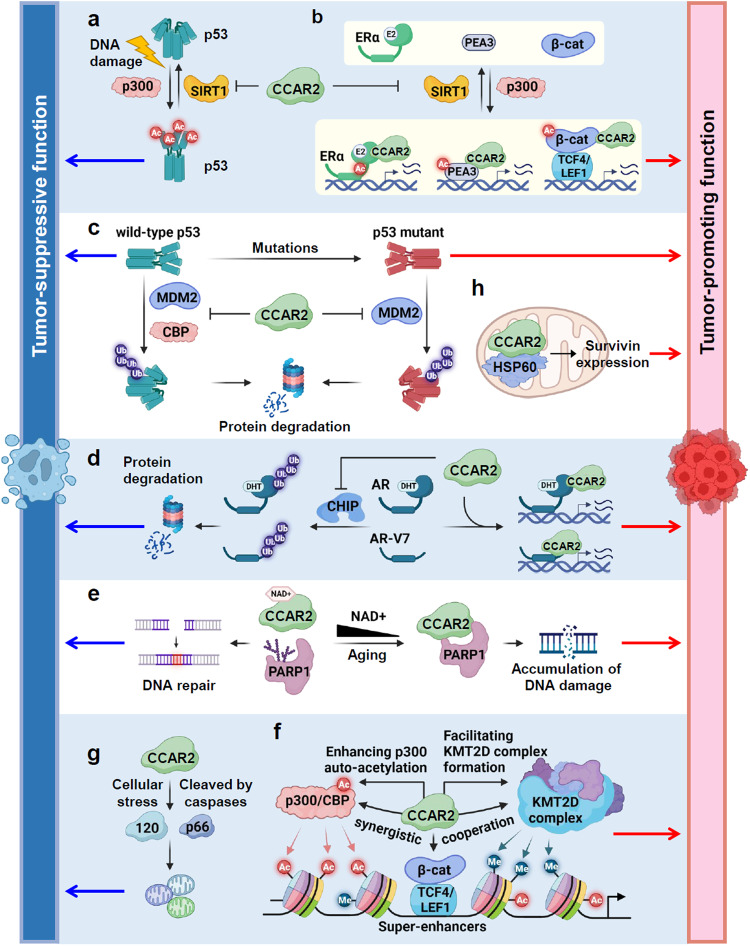
The dual role of CCAR2 in tumorigenesis. **a** CCAR2 acts as an inhibitor of SIRT1-mediated deacetylation and repression of p53 and promotes p53-mediated apoptosis under DNA damage conditions. **b** CCAR2 acts as a coactivator of ERα and PEA3/ETV4 by inhibiting SIRT1 activity. CCAR2 inhibits SIRT1-mediated deacetylation and repression of ERα and PEA3/ETV4 by competing with SIRT1 for binding to ERα and PEA3/ETV4, thereby increasing their DNA-binding and transcriptional activities and promoting breast cancer progression. In addition, CCAR2 interacts with and protects β-catenin from SIRT1-mediated deacetylation by blocking the access of SIRT1 to β-catenin, thereby enhancing LEF1–β-catenin complex formation on chromatin and promoting Wnt/β-catenin-mediated colorectal cancer progression. **c** CCAR2 plays an important role in tumor suppression by directly binding and stabilizing p53. CCAR2 blocks MDM2-mediated ubiquitination and degradation of p53 by competing with MDM2 for p53 binding. CCAR2 also stabilizes mutant p53 and promotes its oncogenic function. Thus, CCAR2 could have tumor-suppressive and tumor-promoting activity depending on p53 genetic status. **d** CCAR2 binds to AR and AR-V7 and inhibits CHIP-mediated AR and AR-V7 ubiquitination by interfering with CHIP binding to AR and AR-V7, thereby increasing their stability and DNA-binding activity and promoting the development and progression of castration-resistant prostate cancer. **e** At high NAD+ levels, NAD+ binds to CCAR2 and inhibits it from interacting with PARP1, which promotes the DNA repair activity of PARP1. A decrease in NAD+ levels during aging promotes the binding of CCAR2 to PARP1, which inhibits PARP1 activity and thus causes the accumulation of DNA damage. **f** CCAR2 is targeted to superenhancers by the Wnt TFs TCF4/LEF1 and β-catenin. CCAR2 promotes p300-mediated H3 acetylation, KMT2D-mediated H3K4 methylation, and their cooperative histone-modifying activities by facilitating their interaction and recruitment to superenhancers, suggesting a critical role for CCAR2 in colorectal cancer progression by regulating colorectal cancer-driven superenhancers and their target gene expression. **g** Under cellular stress conditions, nuclear CCAR2 is processed by caspases to produce CCAR2 p120 and p66 C-terminal fragments. Truncated CCAR2 proteins localize to the cytoplasm, where they promote mitochondrial clustering and sensitize cells to apoptotic cell death. **h** Under mitochondrial stress conditions, the CCAR2-HSP60 interaction is increased in mitochondria, and CCAR2 and HSP60 promote cancer cell survival by upregulating mitochondrial survivin expression.

Early reports on CCAR2 as a negative regulator of the histone deacetylases SIRT1 and HDAC3 and the histone methyltransferase (HMT) SUV39H1 suggested that CCAR2 plays a critical role as a regulator of epigenetic modifying enzymes in the epigenetic regulation of gene expression^7,8,15,17^. SIRT1, the mammalian ortholog of yeast Sir2, is an NAD+-dependent deacetylase belonging to the sirtuin family (SIRT1-7)^47^. SIRT1 catalyzes the deacetylation of histones and numerous nonhistone proteins by consuming NAD+ and plays an important role in multiple biological processes, including metabolism, aging, apoptosis, tumorigenesis, and epigenetic processes^22,47^. SIRT1 contributes to heterochromatin formation and gene silencing by deacetylating histones. In addition, SIRT1 can regulate transcription by deacetylating TFs and coregulators. An increasing number of studies have demonstrated that CCAR2 is a key regulator of SIRT1. In 2008, two parallel studies identified CCAR2 as a negative regulator of SIRT1 and showed that CCAR2 directly interacts with the catalytic domain of SIRT1 using its NTD and thus inhibits the deacetylase activity of SIRT1 against the tumor suppressor TFs p53 and FOXO3a^7,8^ (Fig. 2a). The C-terminal region of SIRT1 includes a 25 amino acid sequence (named ESA [essential for SIRT1 activity]), which interacts with and functions as an activation switch for the catalytic core of SIRT1^48^. CCAR2 inhibits SIRT1 activity by competing with the ESA motif for binding to the catalytic core of SIRT1. The role of CCAR2 as a negative regulator of SIRT1 was further supported by later studies showing that CCAR2 negatively regulates SIRT1-mediated deacetylation of ERα, PEA3/ETV4, and β-catenin^5,9,14^ (Fig. 2b).

The interaction between SIRT1 and CCAR2 is tightly regulated by PTMs. AMPK (AMP-activated protein kinase) binds to the catalytic domain of SIRT1 and phosphorylates SIRT1 at multiple Ser/Thr residues, which promotes SIRT1-CCAR2 dissociation and enhances the deacetylase activity of SIRT1^49,50^. DNA damage and oxidative stress trigger the phosphorylation of CCAR2 on Thr454 by ATM (ataxia telangiectasia-mutated)/ATR (ataxia telangiectasia and Rad3-related) kinases^51,52^. This phosphorylation increases the CCAR2-SIRT1 association, inhibiting SIRT1 activity and enhancing p53 acetylation and transcriptional function. In addition to phosphorylation, CCAR2 is acetylated on Lys112 and Lys215 by hMOF (human males absent on the first), and acetylation inhibits the CCAR2-SIRT1 interaction^37^. Upon DNA damage, the CCAR2 acetylation is reduced in an ATM-dependent manner, increasing the CCAR2-SIRT1 complex formation and p53 acetylation. Interestingly, SIRT1 also promotes deacetylation of CCAR2^37^, suggesting, since CCAR2 deacetylation increases inactive SIRT1-CCAR2 complex formation, the presence of a negative-feedback mechanism that limits SIRT1 deacetylase activity. Genotoxic stress also induces CCAR2 SUMOylation by SUMO2/3, which enhances the CCAR2-SIRT1 interaction and promotes p53-mediated apoptosis^53^. In addition to PTMs, the SIRT1-CCAR2 interaction can be modulated by coregulators or RNA molecules. CCAR1 and BRMS1 (breast cancer metastasis suppressor 1) inhibit CCAR2-SIRT1 complex formation by competing with SIRT1 for binding to CCAR2^9,54^. Similarly, MALAT1, a CCAR2-binding lncRNA, also suppresses the CCAR2-SIRT1 association^42^.

In addition to its inhibitory activity against SIRT1, CCAR2 can also negatively regulates the deacetylase activity of HDAC3 on histone peptides and TF MEF2D (myocyte enhancer factor 2D) and alter subcellular localization by binding to the C-terminal region of HDAC3^15^. Negative regulation of HDAC3 by CCAR2 was confirmed in a later study showing a role for CCAR2 in the regulation of ELL (eleven-nineteen lysine-rich in leukemia) stability^16,55^. ELL is an elongation factor that stimulates transcription elongation by reducing the rate of RNA Pol II stalling. p300-mediated acetylation increases the stability of ELL, whereas deacetylation by HDAC3 decreases its stability through E3 ubiquitin ligase Siah1-mediated polyubiquitination and degradation. CCAR2 competes with HDAC3 for binding ELL and thus increases the acetylation levels and stability of ELL. In addition, proteomics-based interactome studies on human HDACs revealed that CCAR2 can associate with additional deacetylases, HDAC9, HDAC5, and SIRT7^56,57^, suggesting that CCAR2 binds preferentially to certain types of deacetylases and has some specificity as a regulator of deacetylases in cells.

The link between CCAR2 and deacetylases was expanded to another type of epigenetic modifier, SUV39H1^17^. SUV39H1 is an HMT that specifically mediates the trimethylation of histone H3 lysine 9 (H3K9me3) and plays a critical role in heterochromatin formation^58^. SIRT1 interacts with and deacetylates SUV39H1 to stimulate its HMT activity^59^. Thus, the SUV39H1-SIRT1 complex synergistically promotes H3K9me3 deposition and heterochromatin formation. CCAR2 directly binds to the catalytic SET (su(var)3–9, enhancer of zest, and trithorax) domain of SUV39H1 via its N-terminal domain and inactivates the HMT activity of SUV39H1^17^. Moreover, CCAR2 binding to SUV39H1 or SIRT1 disrupts the interaction of SUV39H1 with SIRT1 rather than bridging their interaction. These results suggest that CCAR2 may function as a negative regulator of heterochromatin formation by disrupting the SUV39H1-SIRT1 complex and blocking their histone-modifying activities. As SIRT1, HDAC3, and SUV39H1 interact with and modify histones, TFs, and coregulators, their negative regulator CCAR2 potentially exerts broad effects on the epigenetic regulation of gene transcription.

### Regulation of IKKβ, CHK2, and Aurora B activity by CCAR2

IKKβ (IκB kinase β) is a core component of the signaling cascade of NF-κB proteins, a family of TFs that play essential roles in inflammation, immunity, and cell survival^60^. IKKβ phosphorylates IκB (an inhibitor of NF-κB), targeting it for ubiquitination-mediated degradation, thereby allowing NF-κB to be translocated into the nucleus where it activates its target genes. In addition to IκB phosphorylation, NF-κB signaling is stimulated by the phosphorylation of RelA, a member of the NF-κB family. CCAR2 increases RelA phosphorylation by binding to and enhancing the kinase activity of IKKβ, leading to an increase in NF-κB transcriptional activity and expression of its target genes that are involved in anoikis resistance^61^.

CHK2 and its upstream kinase ATM are essential for sensing DNA damage and triggering the DNA damage response cascade in response to double-strand DNA breaks^62^. Upon DNA damage, ATM phosphorylates CHK2, leading to its autophosphorylation and activation. Activated CHK2 then phosphorylates downstream substrates, including p53, Cdc25C, and KAP1 (KRAB-associated protein 1), and promotes cell cycle arrest, which allows for DNA repair or the induction of apoptosis. CCAR2 directly interacts with the kinase domain of CHK2 and enhances CHK2-mediated phosphorylation of KAP1, a transcriptional corepressor that mediates chromatin compaction and DNA damage-induced chromatin relaxation in a phosphorylation state-dependent manner^63^. These results suggest a possibly important role for CCAR2 in chromatin dynamics following DNA damage. However, the observation that CCAR2 inhibits CHK2 autophosphorylation and Cdc25C phosphorylation^38^ suggests that CCAR2 might differentially regulate the kinase activity of CHK2 in a substrate-dependent manner.

The Aurora B kinase is a component of the chromosomal passenger complex, which is involved in the regulation of kinetochore–microtubule attachment, chromosome segregation, and cytokinesis during mitosis, and its autophosphorylation is essential for its full kinase activity. CCAR2 enhances Aurora B recruitment to the kinetochore, spindle midzone, and equatorial cortex, and promotes its autophosphorylation. CCAR2 deficiency triggers premature loss of cohesion and chromosome decondensation, resulting in aberrant chromosome segregation and cytokinesis, suggesting that CCAR2 plays a crucial role in the mitotic phase of the cell cycle by regulating the activity and recruitment of Aurora B^64^.

### Negative regulation of E3 and E4 ubiquitin ligases by CCAR2

MDM2 (murine double minute 2) is a RING-type E3 ubiquitin ligase that catalyzes the direct transfer of ubiquitin from ubiquitin-charged E2 conjugating enzymes to target proteins, including p53^62,65^. In the absence of cellular stress, MDM2 interacts with p53, inhibits the transcriptional activity of p53 by binding to its N-terminal transactivation domain, and maintains p53 at low steady-state levels by promoting p53 ubiquitination and proteasome-mediated degradation. Multiple lysine residues in the C-terminal regulatory domain of p53 are competitively targeted for either ubiquitination by MDM2 or acetylation by p300/CBP^66^. Under stress conditions, several stress-activated kinases, including ATM/ATR and CHK2, phosphorylate multiple Ser/Thr residues in the N-terminal transactivation domain of p53, leading to the disruption of the p53-MDM2 interaction and increasing the stability and acetylation of p53^65,66^. In addition to the role of CCAR2 as a positive regulator of p53 by inhibiting SIRT1, CCAR2 also stabilizes p53 by inhibiting MDM2-mediated p53 ubiquitination and degradation^67^. In CCAR2-knockout MEFs (mouse embryonic fibroblasts), p53 protein levels, but not mRNA levels, are decreased under stress as well as in normal conditions. Similarly, p53 protein levels are also decreased in the tissues of CCAR2-knockout mice, suggesting that CCAR2 regulates p53 expression at the posttranslational level. CCAR2 interacts with the transactivation and DNA-binding domains of p53, which are the same binding sites for MDM2. CCAR2 blocks p53 ubiquitination by competing with MDM2 for p53 binding and thus stabilizes p53 (Fig. 2c). SIRT1 depletion did not affect p53 protein levels in CCAR2 WT and CCAR2-knockout MEFs^67^, indicating that this function is independent of the role of CCAR2 in the regulation of SIRT1 activity.

p300 and its paralog CBP are general transcriptional coregulators and histone acetyltransferases (HATs) that interact with and regulate the transcriptional activity of multiple TFs^68^. In addition to their acetyltransferase activity, p300/CBP have been shown to possess intrinsic E4 ubiquitin-chain elongation activity that mediates p53 polyubiquitination and degradation in the absence of cellular stress^69,70^. Interestingly, p300/CBP E4 ligase activities are only detected in the cytoplasm, not in the nucleus, suggesting compartment-specific regulation of p300/CBP E4 ligase activities within a cell. A recent proteomic study revealed that CCAR2 stably interacts with CBP in the nucleus and suppresses nuclear p53 polyubiquitination and degradation^71^. Loss of CCAR2 resulted in an increase in nuclear p53-directed CBP E4 activity, and in contrast, restoration of CCAR2 increased p53 levels by inhibiting nuclear p53 polyubiquitination and promoted p53-dependent apoptosis in response to DNA damage. Thus, CCAR2 positively regulates nuclear p53 stability and activity by functioning as a negative regulator of CBP-mediated nuclear polyubiquitination of p53 (Fig. 2c).

CHIP (carboxyl terminal of HSP70-interacting protein) is a RING-like U-box domain-containing E3/E4 ubiquitin ligase that plays a critical role in protein quality control and protein homeostasis by controlling HSP70 (heat shock protein 70)-HSP90 chaperone function between refolding and degradation^72^. CHIP targets a wide variety of proteins, and interestingly, most of its substrates are involved in cancer. CHIP negatively regulates the levels of various oncoproteins (i.e., EGFR, c-Myc, PI3K/AKT, ERα, and AR), resulting in the inhibition of cell proliferation and cancer progression^72^. For example, CHIP binds to the hinge region of AR and increases AR ubiquitination and degradation, leading to the mitotic arrest of prostate cancer cells^73^ (Fig. 2d). Recently, we reported that CCAR2 inhibits the CHIP-mediated ubiquitination and degradation of AR-V7, a constitutively active AR splice variant lacking the ligand-binding domain, as well as AR^13^. The expression of AR-V7 is one of the key mechanisms that promote castration-resistant prostate cancer (CRPC). Depletion or overexpression of CCAR2 decreased or increased, respectively, the protein levels of AR and AR-V7 without affecting their mRNA levels. CCAR2 binds to both CHIP and AR/AR-V7 and inhibits CHIP E3 ligase activity for AR/AR-V7 ubiquitination by blocking the interaction between CHIP and AR/AR-V7 (Fig. 2d). These results indicate that CCAR2 plays a key role in regulating the stability of AR and AR-V7 by functioning as a negative regulator of CHIP.

### Negative regulation of PARP1 by CCAR2

PARP1 is an NAD + -dependent enzyme that catalyzes the transfer of ADP-ribose from NAD+ to substrate proteins and generates poly(ADP-ribose) chains (PARylation), and it is critical to the DNA damage response, DNA repair, apoptosis, and the epigenetic regulation of gene expression^74^. Li et al. showed that NAD+ regulates protein‒protein interactions by binding to the NHD of CCAR2^45^. At low levels of NAD+, CCAR2 binds to PARP1 and inhibits the PARylation activity of PARP1 (Fig. 2e). Indeed, in old mice, whose NAD+ levels decline during aging, CCAR2 increasingly bound to PARP1, leading to increased DNA damage, which was reversed when cellular NAD+ levels were restored. In addition, CCAR2-knockout mice showed increased PARP1 activity, indicating that CCAR2 is a negative regulator of PARP1 and suggesting a link between CCAR2 and age-associated genome instability (Fig. 2e).

### Positive regulation of enhancer epigenomic writers, p300/CBP, and KMT2D, by CCAR2

PTMs, such as acetylation and methylation, of histone tails, cooperatively regulate chromatin accessibility and gene transcription^58,75,76^. The epigenetic writers p300/CBP and KMT2D play pivotal roles in establishing and maintaining active enhancer and promoter states enriched with acetylated histone H3K27 (H3K27ac) and monomethylated H3K4 (H3K4me1) or trimethylated H3K4 (H3K4me3)^76^. Genome-wide studies have identified superenhancers that drive the expression of genes to control cell identity and promote oncogenic transcription^76,77^. Superenhancers are large clusters of enhancers that are co-occupied by many TFs and coactivators and are extensively marked by H3K27ac (a marker of superenhancers)^30,76,77^. Recent work by our group showed that CCAR2 plays a critical role in establishing active chromatin landscapes and superenhancers in colon cancer cells by regulating p300- and KMT2D-mediated epigenetic modifications of histone H3^19^ (Fig. 2f).

The transcriptional coactivators p300/CBP are HATs that regulate gene expression by acetylating a variety of protein substrates, including core histones, TFs, and coregulators^68^. CCAR2 specifically binds to the HAT domain of p300/CBP via its NTD and greatly enhances p300/CBP-mediated histone H3 acetylation, such as H3K4ac and H3K27ac^19^ (Fig. 2f). Interestingly, CCAR2 also enhances p300 HAT autoacetylation^19^, which leads to an increase in p300 HAT activity^78^, indicating that CCAR2 positively regulates p300/CBP catalytic activity by enhancing their autoacetylation (Fig. 2f).

KMT2D, also known as MLL4 (mixed-lineage leukemia 4), belongs to the KMT2 family of proteins which catalyze H3K4 methylation (H3K4me1/2/3) and functions in multisubunit complexes with WDR5, RBBP5, ASH2L, and DPY30 as core components that are required for the efficient catalytic activity of KMT2D^58,79^. KMT2D has a conserved SET domain in its C-terminus, which is the catalytic domain responsible for HMT activity. KMT2D is known as a major H3K4me1/2 methyltransferase and has weak enzyme activity for H3K4me3^79,80^. Interestingly, CCAR2 enhances the overall catalytic activity of KMT2D for H3K4me1/2/3^19^, suggesting that CCAR2 can promote the rate and processivity of KMT2D enzyme activity. CCAR2 loss and overexpression impairs and increases the association of KMT2D with its core components, respectively, indicating that CCAR2 enhances KMT2D activity by promoting the assembly of the KMT2D complex (Fig. 2f).

As major enhancer writers, KMT2D and p300 are enriched at active enhancers, including superenhancers, and are reciprocally required for efficient chromatin binding^81,82^. CCAR2 promotes the cooperative effects of KMT2D and p300 on H3K4 methylation and H3 acetylation by increasing the interaction between the catalytic domains of KMT2D and p300^19^ (Fig. 2f). Furthermore, loss of CCAR2 results in the global redistribution of KMT2D and p300 genomic binding and decreases KMT2D and p300 occupancy at CCAR2-regulated enhancers and thus reduces their target gene expression. These results indicate that CCAR2 contributes to active enhancer-associated histone modifications by enhancing the histone-modifying activities of KMT2D and p300 and facilitating their enhancer recruitment and functional cooperation (Fig. 2f).

## Dual function of CCAR2 as a tumor suppressor and tumor promoter

### Tumor suppressor function of CCAR2

A genetic screen identified CCAR2 as a gene homozygously deleted in breast cancer and some other tumors, postulating that CCAR2 is a potential tumor suppressor^3^. As its gene name CCAR2 indicates, initial studies showed that CCAR2 is involved in cell cycle regulation and apoptosis^83^. During tumor necrosis factor α- and DNA damage-induced apoptosis, CCAR2 undergoes caspase-dependent cleavage, generating C-terminal p120 and p66 fragments that lack an N-terminal NLS. The truncated forms of CCAR2 then localize from the nucleus to the cytoplasm, inducing mitochondrial clustering and sensitizing cells to apoptotic cell death^83^ (Fig. 2g). These results suggest that CCAR2 loss or mutations rendering resistance to processing may induce resistance to apoptosis and thus promote tumor growth and progression. Subsequently, two independent studies identified CCAR2 as a SIRT1-binding protein and demonstrated that the CCAR2-SIRT1 interaction inhibits SIRT1 deacetylase activity and promotes the acetylation of p53 and FOXO3 following DNA damage, resulting in the activation of p53-dependent apoptosis in several cancer cell lines^7,8^ (Fig. 2a). Notably, CCAR2-depleted cells were resistant to apoptosis, and concomitant depletion of SIRT1 reversed the inhibitory effects of CCAR2 knockdown on p53-mediated apoptosis. In addition, hypoxia-induced ubiquitination and degradation of CCAR2 by Siah2 promoted breast cancer cell proliferation and inhibited DNA damage-induced apoptosis by decreasing p53 acetylation, further supporting a tumor-suppressive role for CCAR2 in cancer^84^. Furthermore, although SIRT1 enhances c-MYC-driven tumorigenesis, CCAR2 promotes c-MYC-induced apoptosis by inhibiting SIRT1 activity in several different cell lines^85^. These results demonstrate that CCAR2 plays a key role as a tumor suppressor by activating the p53-, FOXO3-, and c-MYC-mediated apoptotic pathways via its inhibition of the tumor-promoting functions of SIRT1.

CCAR2 also activates p53-dependent apoptosis mediated through SIRT1-independent mechanisms. CCAR2 stabilizes p53 by competing with MDM2 for p53 binding and by inhibiting the nuclear p53-directed E4 activity of CBP, leading to interference with MDM2- and CBP-mediated polyubiquitination and degradation of p53 and enhancing p53-dependent apoptosis^67,71^ (Fig. 2c). Loss of CCAR2 induced binucleation in MEFs and promoted cell proliferation and transformation^67^. Moreover, CCAR2-knockout mice were more susceptible to tumor development, and their disease-free survival was significantly lower than that of wild-type mice^67^. However, CCAR2/p53 double-knockout in mice did not further affect the survival rate and tumor incidence, suggesting that CCAR2 suppressed tumorigenesis mainly through p53 in vivo. Intriguingly, a pancancer analysis based on TCGA (The Cancer Genome Atlas) revealed that CCAR2 gene alterations are generally associated with the retention of wild-type p53 in several cancer types^71^. For example, 30% of breast cancer and 96% of prostate cancer cases with CCAR2 deletion maintain wild-type p53 status. This strong association between CCAR2 loss and the maintenance of wild-type p53 activity suggests that CCAR2 loss may lead to p53 pathway inactivation. Together, these results indicate that CCAR2 functions as a tumor suppressor mainly by regulating the transcriptional activity and stability of p53.

### Role of CCAR2 as a tumor-promoting coactivator

The role of CCAR2 in tumorigenesis has been debated. CCAR2 has been reported to be downregulated or upregulated and to serve as either an indicator of good or poor prognosis in various cancers, even in the same type of cancer, including breast cancer^6,20,21^ (Table 2). In addition, the p53 gene (TP53) is the most frequently mutated gene in human cancers. Cancer genome sequencing studies showed that 42% of patients in a pancancer cohort harbored mutations in the TP53 gene^86^. Tumors harboring TP53 mutations progress more rapidly and are more resistant to anticancer therapy than tumors harboring wild-type p53^87^, indicating that p53 mutants not only lose their tumor suppressor effects but also acquire new oncogenic activities that promote cancer progression. Thus, TP53 mutation status in cancer cells may be related to the conflicting descriptions of CCAR2 role in tumorigenesis. Indeed, CCAR2 stabilizes the oncogenic p53 R280K mutant in cancer cells by binding to and blocking ubiquitination of the mutant p53^67^ (Fig. 2c). In addition, CCAR2 loss in cancer cells harboring mutant p53 decreases cancer cell proliferation and increases sensitivity to chemotherapy^67^. Furthermore, in a TCGA analysis, glioma patients with high CCAR2 expression in combination with TP53 mutations showed reduced relapse-free survival than patients with low CCAR2 and TP53 mutations^6^. In contrast, in conjunction with wild-type TP53 expression, high CCAR2 expression is associated with better survival. These results indicate that CCAR2 functions as a tumor suppressor or a tumor promoter depending on the TP53 mutation status of cancer cells.

**Table 2:** Changes in CCAR2 expression levels associated with tumor progression.

Cancer type	Expression level associated with poor prognosis	Ref
Breast cancer	Upregulated	^4,5^
Colorectal cancer	Upregulated	^14,95,96^
Esophageal squamous cell carcinoma	Upregulated	^111^
Hepatocellular carcinoma	Upregulated	^112,113^
Osteosarcoma	Upregulated	^92^
Ovarian cancer	Upregulated	^114^
Soft tissue sarcoma	Upregulated	^115^
Clear cell renal cell carcinoma	Upregulated	^116^
Diffuse large B cell lymphoma	Upregulated	^117^
Gastric cancer	Upregulated	^89,118^
	Downregulated	^119^
Pancreatic cancer	Downregulated	^120^
Bladder cancer	Downregulated	^121^
Gallbladder carcinoma	Downregulated	^122^
Laryngeal & hypopharyngeal carcinoma	Downregulated	^123^

The complexity of the CCAR2 roles in tumorigenesis can also be attributed to its ability to interact with diverse proteins and regulate their cellular functions. CCAR2 interacts with nuclear receptors, such as ERα^9,10^, ERβ^32^, AR^12,13^, COUP-TF1^33^, Rev-Erbα^46^, RARα^88^, and LXRα^34^, and other types of oncogenic TFs, such as c-Myc^85^, PEA3/ETV4^5^, and NF-κB^35,61,89^, to regulate their transcriptional activity (Table 1). ERα and AR are ligand-dependent TFs that mediate the diverse biological effects of estrogens and androgens, respectively, including the maintenance of normal physiology and development of hormone-dependent cancers^90^. ERα and AR are acetylated and deacetylated by p300 and SIRT1, respectively, and their acetylation enhances their DNA-binding and transcriptional activity^91^. CCAR2 is required for estrogen-stimulated growth of ERα-positive breast cancer cells as well as estrogen-induced ERα target gene expression^9^. The mechanism underlying CCAR2 action involves the inhibition of SIRT1-mediated deacetylation and repression of ERα activity. CCAR2 represses SIRT1 activity by blocking the interaction of SIRT1 with its substrate ERα and consequently enhances ERα recruitment to its target enhancers (Fig. 2b). Moreover, the coactivator activity of CCAR2 is further enhanced by Ajuba (ajuba LIM protein), which promotes CCAR2-p300/CBP complex formation and thus increases ERα acetylation^11^. In addition, CCAR2 stabilizes unliganded ERα and suppresses apoptosis in breast cancer cells^10^. Interestingly, CCAR2 and its paralog CCAR1 enhance ERα activity in a synergistic manner, and SIRT1 represses this coactivator synergic effects in a deacetylase activity-independent manner by competing with CCAR1 for CCAR2 binding^9^. These results suggest that, in contrast to its tumor-promoting role in the regulation of p53 activity, SIRT1 represses oncogenic estrogen signaling in breast cancer cells, both directly and indirectly, by deacetylating ERα and disrupting coactivator complex assembly. Moreover, CCAR2 also functions as a coactivator for the oncogenic TF PEA3/ETV4 and promotes ERα-negative breast cancer cell growth, tumorigenesis, and cancer progression^5^. CCAR2 inhibits SIRT1-mediated PEA3/ETV4 deacetylation and thus increases the DNA-binding and transcriptional activity of PEA3/ETV4 (Fig. 2b). CCAR2 depletion reduces the tumorigenic potential of ERα-negative breast cancer cells^5^, and increased expression of CCAR2 has been associated with worsened relapse-free survival of ERα-positive and ERα-negative breast cancer patients^4,5^. These results indicate that CCAR2 plays critical oncogenic roles in both ERα-positive and ERα-negative breast cancers.

As an AR coactivator, CCAR2 promotes AR transcriptional activity by enhancing its DNA-binding activity and its stability in prostate cancer and osteosarcoma cells^12,13,92^. In addition, CCAR2 contributes to CRPC progression by stabilizing and activating AR-V7 by inhibiting the CHIP-mediated ubiquitination and degradation of AR-V7^13^ (Fig. 2d). CCAR2 depletion suppresses the tumorigenic and metastatic potential of CRPC cells, suggesting a tumor-promoting role for CCAR2 in the development and progression of both androgen-sensitive prostate cancer and CRPC. Further studies are needed to explore the possibility that CCAR2 and SIRT1 reciprocally regulate AR and AR-V7 activities by modulating their acetylation status.

CCAR2 also plays an integral role as a key coactivator in Wnt/β-catenin-mediated colorectal cancer (CRC) progression. Upon Wnt signaling activation, β-catenin is stabilized by escaping from phosphorylation/ubiquitination-mediated degradation and regulates Wnt target gene expression by functioning as a coactivator for Wnt-regulated TFs TCF4 (T-cell factor 4) and LEF1 (lymphoid enhancer factor 1)^93^. β-catenin acetylation, which is regulated by p300 and SIRT1^93^, enhances its coactivator function by increasing the interaction with TCF4/LEF1^94^. CCAR2 stabilizes the LEF1-β-catenin interaction by inhibiting SIRT1-mediated deacetylation of β-catenin by blocking their interaction, thereby enhancing LEF1-β-catenin complex formation on Wnt responsive enhancers and increasing the expression of Wnt/β-catenin target genes, including cancer-promoting TFs PROX1 and MACC1^14,95^ (Fig. 2b). Furthermore, CCAR2 functions as a coactivator of PROX1 and MACC1^14,95^, which are reported to promote CRC progression and metastasis, suggesting that CCAR2 plays a key role in CRC progression not only by enhancing LEF1-β-catenin-mediated transcription but also by amplifying Wnt/β-catenin signaling via activating Wnt/β-catenin-inducible TFs. Importantly, CCAR2 is required for tumor growth, metastatic potential, and cancer stem cell-like properties of colon cancer cells, and increased expression of CCAR2 has been associated with poor prognosis and short disease-free survival of CRC patients^14,95,96^.

In addition, CCAR2 has been reported to be required for the antiapoptotic activity of cancer cells. CCAR2 deficiency promotes DNA damage-induced apoptosis in breast and non-small cell lung cancer cells^97,98^ and induces cell death in anti-estrogen-resistant breast cancer cells^99^. CCAR2 also protects cancer cells from mitochondrial oxidative stress-induced apoptosis by activating the expression of mitochondrial survivin by interacting with HSP60, a prosurvival chaperone in mitochondria^100,101^ (Fig. 2h). Furthermore, CCAR2 suppresses anoikis, a special type of apoptosis induced by the loss of cell–matrix contact, and induces anoikis resistance, a key characteristic of metastatic cancer cells, in breast and gastric cancer cells by activating the IKK-β/NF-κB signaling pathway and enhancing the expression of NF-κB target genes involved in anoikis resistance^61,89^. These results suggest that CCAR2 plays a critical role in the apoptosis resistance of cancer cells under various cellular stress conditions.

### Tumor-promoting role of CCAR2 in the regulation of chromatin structure and epigenetic histone modifications

SUV39H1 is an HMT responsible for the establishment of H3K9me3 at the pericentric region, and reduced H3K9me3 abundance at the pericentric heterochromatin results in genomic instability, a hallmark of cancer^58^. Thus, the discovery of CCAR2 as an inhibitor of the tumor suppressor SUV39H1 suggested that CCAR2 may be a critical regulator of heterochromatin formation and genome stability and, thus, a contributor to heterochromatin relaxation at the pericentric region, possibly playing a role leading to tumorigenesis^17^. KLLN (killin), a tumor suppressor that enhances SUV39H1 HMT activity, promotes H3K9me3 deposition at the pericentric region by interfering with the inhibitory effect of CCAR2 on SUV39H1^18^. These results support a role for CCAR2 in the relaxation of pericentric heterochromatin and suggest that KLLN is a negative regulator of CCAR2 function.

Enhancers can be located tens to hundreds of kb away from their target genes. The three-dimensional organization of chromatin enables physical communication between distal enhancers and target gene promoters by forming chromatin loops^77^. We have shown that CCAR2 facilitates long-range chromatin interactions between distal enhancers and promoters of tumor-promoting genes such as PROX1, MACC1, and CDH2 in cancer cells^13,14,95^, suggesting that CCAR2 activates oncogenic gene transcription by regulating chromatin architecture. Our recent findings expanded the role of CCAR2 to include regulation of the chromatin landscape and function^19^. We showed that CCAR2 is required for establishing the active chromatin landscape and for epigenetic regulation of histone modifications in CRC cells. Loss of CCAR2 resulted in genome-wide dysregulation of active chromatin marks, alterations in the active enhancer landscape, and downregulation of gene expression programs involved in CRC progression and metastasis, such as the Wnt/β-catenin pathway and epithelial-mesenchymal transition. Superenhancers drive the expression of genes that control cell identity and promote cancer progression^30,77,102,103^. Cancer cells frequently acquire superenhancers, and cancer-specific superenhancers function as key drivers of dysregulated gene expression in various cancers. For example, CRC-acquired superenhancers are closely associated with oncogenes and enriched with TCF4/LEF1-binding motifs; TCF4/LEF1 are the terminal TFs involved in the oncogenic Wnt/β-catenin signaling pathway^104^. Interestingly, CCAR2 contributes to superenhancer formation and function in CRC cells not only by facilitating the recruitment of the enhancer epigenomic writers KMT2D and p300 but also by enhancing their functional interaction and cooperativity^19^ (Fig. 2f). CCAR2-regulated superenhancers are enriched with TCF4/LEF1-binding motifs, and their target genes are enriched in pathways associated with CRC progression and are highly expressed in CRC tissues compared with normal colon tissues. Together, these results suggest that CCAR2 plays a crucial role in cancer progression by regulating chromatin structure and function to establish a favorable epigenetic environment for cancer-specific gene expression.

The tumor-promoting role of CCAR2 is further supported by recent meta-analyses^105,106^. Upregulation of CCAR2 is associated with short overall survival and relapse-free survival in various human cancers, suggesting that CCAR2 can be used as a prognostic marker for the survival of cancer patients and as a novel target for cancer therapy.

## Conclusions and perspectives

More than 15 years after the identification of CCAR2 as an inhibitor of SIRT1, many efforts have been made to understand the molecular mechanisms underlying CCAR2 function in the regulation of normal and pathological cellular processes. As discussed above, CCAR2 functions as a key regulator of epigenetic modifiers and TFs in various biological processes, such as apoptosis, DNA repair, and tumor progression. The conflicting roles of CCAR2 in tumorigenesis, in which it shows opposite functions, underscores the complexity of the cellular function of CCAR2 under physiological and pathological conditions. Possible factors underlying this complexity may include TP53 mutation status and the ability of CCAR2 to interact with multiple proteins involved in apoptotic and oncogenic signaling pathways. In addition, an increasing number of studies have revealed a link between CCAR2 and metabolism and circadian clocks^21,23,41,46,107–109^. Because the dysregulation of these processes has been also associated with cancer progression, further research on the role of CCAR2 in cancer-associated metabolism and circadian disruption is needed to understand the complex function of CCAR2 in tumorigenesis and to provide novel mechanistic insights into the therapeutic potential of targeting CCAR2 in cancer treatment. Although CCAR2 has been reported to be either upregulated or downregulated in cancers^6,21^, it is still unclear how CCAR2 gene transcription is differentially regulated in different types of cancer. Thus, to further establish CCAR2 as a therapeutic target for the treatment of cancers, it is important to understand how its expression and activity are regulated in cancer cells and to investigate whether there are genetic mutations in the CCAR2 gene that cause loss- and/or gain-of-function.

Despite an increasing understanding that coregulators act as major contributors to a wide range of diseases, many coregulators remain outside the reach of pharmacological intervention due to the lack of a high-affinity ligand-binding pocket or a defined catalytic surface. The recent finding that NAD+ binding to the NHD of CCAR2 prevents this protein from binding to and inhibiting PARP1^45^ suggests that the CCAR2-NHD may form a ligand-binding pocket and that CCAR2 function might be regulated by ligand binding. Given that recent evidence suggests that CCAR2 is a multifunctional regulator of physiological and pathological cellular processes, identifying potent and selective small molecules targeting CCAR2 is a promising therapeutic strategy for transcriptionally and epigenetically dysregulated cancers and opens many new therapeutic opportunities in a variety of human diseases.
